# Identification ferroptosis-related hub genes and diagnostic model in Alzheimer’s disease

**DOI:** 10.3389/fnmol.2023.1280639

**Published:** 2023-10-30

**Authors:** Huabin Zhao, Jiawei Wang, Zhongzheng Li, Shenghui Wang, Guoying Yu, Lan Wang

**Affiliations:** State Key Laboratory of Cell Differentiation and Regulation, Henan International Joint Laboratory of Pulmonary Fibrosis, Henan Center for Outstanding Overseas Scientists of Pulmonary Fibrosis, College of Life Sciences, Institute of Biomedical Science, Henan Normal University, Xinxiang, Henan, China

**Keywords:** Alzheimer’s disease, ferroptosis, diagnosis biomarkers, immune infiltration, machine learning

## Abstract

**Background:**

Ferroptosis is a newly defined form of programmed cell death and plays an important role in Alzheimer’s disease (AD) pathology. This study aimed to integrate bioinformatics techniques to explore biomarkers to support the correlation between ferroptosis and AD. In addition, further investigation of ferroptosis-related biomarkers was conducted on the transcriptome characteristics in the asymptomatic AD (AsymAD).

**Methods:**

The microarray datasets GSE118553, GSE132903, GSE33000, and GSE157239 on AD were downloaded from the GEO database. The list of ferroptosis-related genes was extracted from the FerrDb website. Differentially expressed genes (DEGs) were identified by R “limma” package and used to screen ferroptosis-related hub genes. The random forest algorithm was used to construct the diagnostic model through hub genes. The immune cell infiltration was also analyzed by CIBERSORTx. The miRNet and DGIdb database were used to identify microRNAs (miRNAs) and drugs which targeting hub genes.

**Results:**

We identified 18 ferroptosis-related hub genes anomalously expressed in AD, and consistent expression trends had been observed in both AsymAD The random forest diagnosis model had good prediction results in both training set (AUC = 0.824) and validation set (AUC = 0.734). Immune cell infiltration was analyzed and the results showed that CD4+ T cells resting memory, macrophages M2 and neutrophils were significantly higher in AD. A significant correlation of hub genes with immune infiltration was observed, such as DDIT4 showed strong positive correlation with CD4+ T cells memory resting and AKR1C2 had positive correlation with Macrophages M2. Additionally, the microRNAs (miRNAs) and drugs which targeting hub genes were screened.

**Conclusion:**

These results suggest that ferroptosis-related hub genes we screened played a part in the pathological progression of AD. We explored the potential of these genes as diagnostic markers and their relevance to immune cells which will help in understanding the development of AD. Targeting miRNAs and drugs provides new research clues for preventing the development of AD.

## Introduction

As the primary cause of dementia, AD is a progressive neurodegenerative disease associated with aging (Ambrogio et al., 2019). Its core pathological features include the accumulation of amyloid-β protein and hyperphosphorylated tau (Surguchov et al., 2023). According to the standards of the National Institute on Aging, NIH and the Alzheimer’s Association, the appearance of AD pathology may occur without any symptoms (Knopman et al., 2019). It then progresses to mild cognitive impairment, with a decline in learning and memory abilities, and ultimately to significant dementia and loss of the ability to live independently. This span can last for 15–25 years, but not every patient will follow this continuous process (Scheltens et al., 2021). An assessment report on survival after dementia diagnosis in the United States indicated a survival time of only 3–4 years (Jack et al., 2019). Similarly, a study of another European cohort showed a median survival time of 6 years after diagnosis (Vermunt et al., 2019). The sharp increase in AD patients has brought heavy pressure to families and society. Based on a highly complex process that integrates behavioral, genetic, and environmental factors, extensive research has been conducted on the pathogenesis of AD.

Interestingly, in addition to the deposition of amyloid-β protein and tau, the brains of AD patients also exhibit progressive neuronal loss and oxidative stress caused by metal homeostasis imbalance (Plascencia-Villa and Perry, 2021). With the discovery of ferroptosis - a form of programmed cell death mediated by lipid peroxidation that is distinct from autophagy, apoptosis, and necrosis (Dixon et al., 2012; Jiang et al., 2021) – the hypothesis of transitional metal imbalance has gained strong support, and researchers believe that ferroptosis may be an important cause of neuronal loss in AD patients. Iron metal is involved in critical processes such as myelin formation, neuronal activity, neurotransmitter synthesis, and energy metabolism in the brain (Crichton et al., 2011). Its storage, distribution, and efflux are precisely regulated by transcriptional levels of iron response elements and iron regulatory proteins (Ward et al., 2014). Studies have shown that iron homeostasis imbalance can block the activity of the mitochondrial electron transport chain, causing oxidative stress (Onukwufor et al., 2022). In summary, ferroptosis plays an important role in AD pathology. Moreover, recent reports have also pointed to the pathogenesis of AD to lysosomal autophagy and neuroinflammation (Calsolaro and Edison, 2016; Lee et al., 2022). Peripheral immune cells, such as bone marrow-derived monocytes, are recruited into the brain to efficiently clear amyloid deposits (Simard et al., 2006). On the other hand, researchers found CD8 + T cells in the hippocampus of AD patients after death, which may be one of the direct causes of neuronal dysfunction (Unger et al., 2020). Therefore, this study will also focus on the immune penetration in AD, to analyze the composition of immune cells.

MicroRNAs are small RNA molecules which associated with many neurodegenerative diseases that regulate gene expression by binding to target mRNAs (Juźwik et al., 2019). Previous studies have reported the abnormal expression of hsa-miR-4286 in AD (Henriques et al., 2020) and its association with Parkinson’s disease neurodegeneration (Su et al., 2018). MicroRNAs (miRNAs) and their target genes are intensely studied as candidates for diagnostic and prognostic biomarkers. For instance, in the cerebrospinal fluid of AD patients, exosomal miR-193b was negatively correlated with amyloid-β (Liu et al., 2014). In this study, we aimed to screen abnormally expressed ferroptosis-related genes in AD and identify miRNAs with diagnostic or therapeutic potential by targeting ferroptosis-related genes. Diagnostic models were constructed through hub genes to judge the potential of these genes to identify AD, while assessing the correlation of these genes with immune infiltration in AD patients. Finally, by screening the MicroRNAs and drugs targeting hub genes, we can provide valuable data support for the clinical treatment and drug development of AD.

## Materials and methods

### Dataset download

The microarray datasets GSE118553, GSE132903, GSE33000 and GSE157239 on AD were downloaded from the Gene Expression Omnibus (GEO) database^1^ (Barrett et al., 2013). The annotation information of the chip probes of the corresponding platforms was obtained from the GEO database, respectively. We collected clinical features from various datasets, including age, gender and the number of individuals in each group (Table 1).

**Table 1:** Microarrays datasets clinical characteristics.

Dataset	GSE118553			GSE132903		GSE33000		GSE157239	
Groups	Control	AsymAD	AD	Control	AD	Control	AD	Control	AD
Number	100	134	167	98	97	157	310	8	8
Age	70.44 ± 15.79	86.28 ± 8.59	82.92 ± 10.20	84.98 ± 6.90	85.02 ± 6.75	63.52 ± 9.91	80.60 ± 8.99	79.88 ± 7.32	79.75 ± 9.66
Gender									
Male	55	40	69	50	49	123	135	3	2
Female	45	94	98	48	48	34	175	5	6

### Differential expression and pathways enrich analysis

The R software package limma (Ritchie et al., 2015) was used to achieved differential analysis on control and AD samples. Genes with p_adj < 0.05 and abs(logFC) > 0.585 were considered as DEGs. Heat maps and bot plots of DEGs were created using the “pheatmap” and “ggplot2” packages. Kyoto encyclopedia of gene and genomes (KEGG) pathway enrichment analysis were performed on DEGs to explore the biological functions and related pathways of DEGs.

### Ferroptosis-related genes acquisition

The list of ferroptosis-related genes was extracted from the FerrDb website (Zhou et al., 2023). By intersecting the ferroptosis-related genes list with DEGs, finally 18 ferroptosis-related hub genes were identified. An interaction network for the ferroptosis-related genes was generated by the STRING database (Szklarczyk et al., 2011).

### Diagnostic model construction

The 18 ferroptosis-related hub genes were used to constrct the random forest model. To further obtain the error-stable model, appropriate parameters were selected by varying the number of decision trees, and the 1,000 trees were finally set as the optimal parameters of the model. Two hundred and sixty-seven samples from GSE118553 were randomly divided into a training set and a testing set using a ratio of 4:1. The importance of features by calculating the purity of nodes through Gini coefficient method were computed.

### Gene set enrichment analysis

Gene set enrichment analysis (GSEA) was used for pathway enrichment analysis (Subramanian et al., 2005). The Molecular Signatures Database (MSigDB) of hallmark gene sets (H), curated gene sets (H2), and GO gene sets (C5) were used for enrichment analysis. An FDR value of 0.05 was used as a cutoff.

### Immune infiltration analysis

CIBERSORTx is an analytical tool to assess the abundance of immune cell subsets in tissue samples by using a deconvolution algorithm (Newman et al., 2019). The LM 22 signature matrix file, which contains 22 immune cell components, was used as a reference for cell quantification. Correlation between immune cells and genes was analyzed by Pearson correlation analysis.

### Exploration microRNAs targeting the hub genes

Differential analysis of the GSE157239 expression matrix was performed using the Limma R package to obtain differentially expressed miRNAs. MiRNAs with p.value <0.05 and abs(logFC) > 0.263 were considered as DEmiRNAs. Potential miRNAs of hub genes were retrieved by miRNet database (Chang et al., 2020). The association between microRNAs and AD was investigated through the HMDD database (Cui et al., 2023).

### Drugs and genes interaction scores

Drugs targeting hub genes were retrieved from the Drug-Gene Interaction Database (DGIdb). The DGIdb is a web resource that provides information on drug-gene interactions and druggable genes from publications, databases, and other web-based sources (Freshour et al., 2021). The bar plots of interaction scores were created using “ggplot2” package.

### Statistical analysis

R software was used for statistical analysis. Principal Component Analysis (PCA) was performed using an R package “factoextra.” The association between continuous variables was assessed using Pearson’s correlation coefficient. *p < 0.05; **p < 0.01; and ***p < 0.001 are considered significant.

## Results

### Identification of DEGs associated with ferroptosis in AD

**Figure 1:**
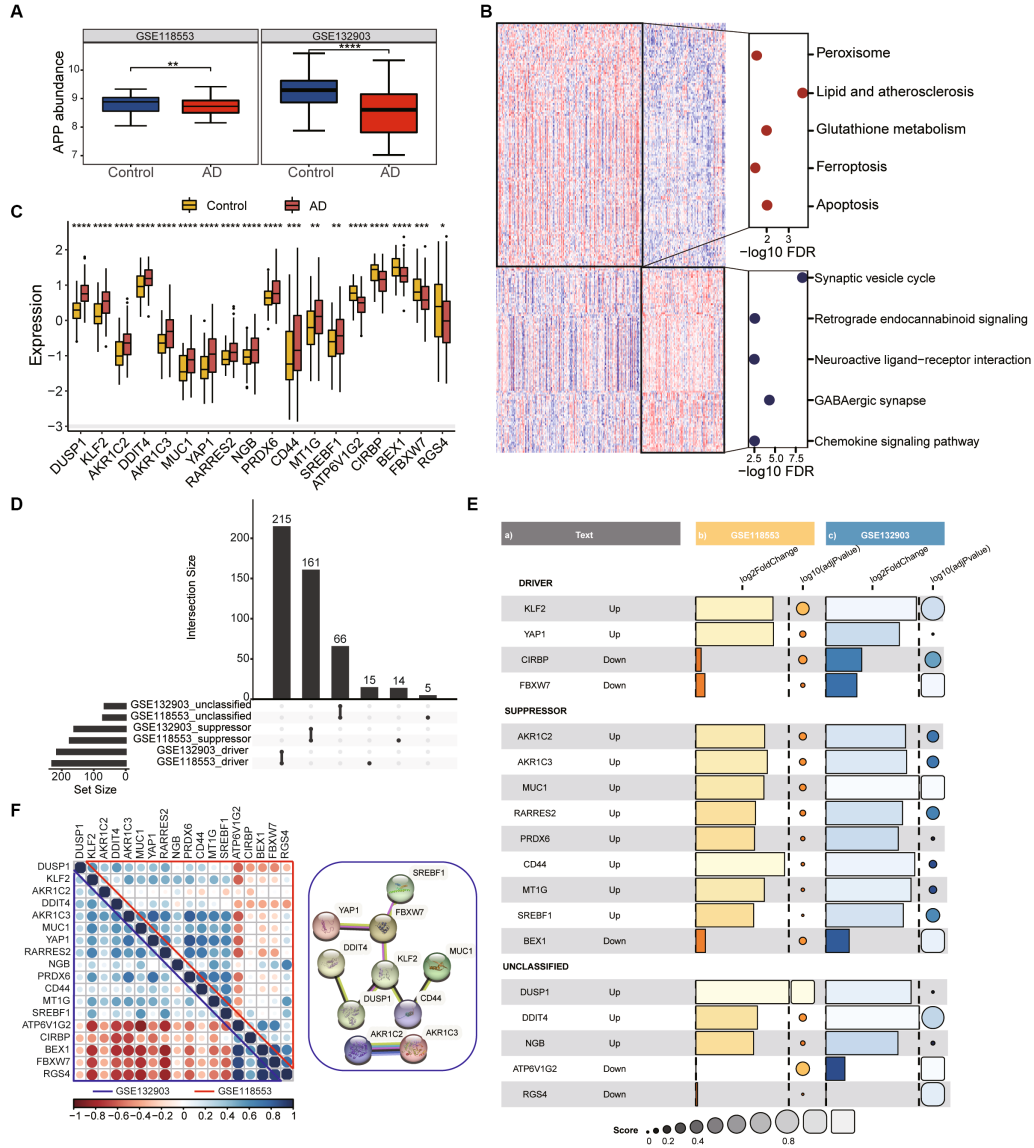
Identification of DEGs associated with ferroptosis in AD. **(A)** The boxplot shows the expression difference of APP between the Control group and AD group. Data were analyzed by Kruskal−Wallis test. **p < 0.01, ****p < 0.0001. **(B)** The heatmap plot shows DEGs between frequent exacerbators and non-frequent exacerbators. Dotplot displays KEGG enrichment analysis of over-represented genes in control and AD groups. **(C)** The expression patterns of ferroptosis-related genes were presented in the boxplot in GSE118553. Data were analyzed by Kruskal−Wallis test. *p < 0.05, **p < 0.01, ***p < 0.001. **(D)** The collection diagram shows driving, suppressing, and unclassified ferroptosis related genes overlapping in the two datasets. **(E)** Ferroptosis related genes with the same expression trend were found in both datasets. Barplot and dotplot show their log2FC and-log10 adjust pvalue. **(F)** Correlation dotplot shows correlations between genes in two datasets. The upper triangle represents GSE118553 and the lower triangle represents GSE132903. Red dots means negative correlation, blue dots means positive correlation. The graph on the right shows part of the interactions between genes.

To screen out the genes associated with ferroptosis that are robustly expressed in AD, two datasets (GSE118553 and GSE132903) were used for the analysis. The expression of amyloid precursor protein (APP) was downregulated in both two datasets (Figure 1A). Firstly, we identified differentially expressed genes (DEGs) between Alzheimer’s disease (AD) and healthy control groups in GSE118553 (Supplementary Figure S1A). We found that upregulated genes in AD were mainly associated with apoptosis and ferroptosis, while downregulated genes were mainly involved in neuroactive ligand-receptor interaction (Figure 1B). To further determine hub genes associated with ferroptosis in AD, we used FerrDB to identify ferroptosis-related genes that were aberrantly expressed in AD (Figure 1C). According to FerrDB, we classify ferroptosis regulators into three categories: Driver, Suppressor, and Unclassified genes. Furthermore, we observed consistent expression trends with GSE118553 of these genes in AD in GSE132903 (Figure 1D) (Supplementary Figures S1B,C) and found significant downregulation of Driver regulators such as KLF2 and YAP1 in AD, as well as significant upregulation of multiple Suppressor regulators (Figure 1E). Correlation analysis revealed that the hub genes exhibit strong associations in both the GSE132903 and GSE118553, additionally, protein–protein interaction network also indicates the interrelated interactions among the hub genes (Figure 1F). The results indicate that we identified a robustly differentially expressed gene list associated with ferroptosis in AD by screening through GSE132903 and GSE118553.

### Machine-learning-based for constructing diagnostic model of AD

**Figure 2:**
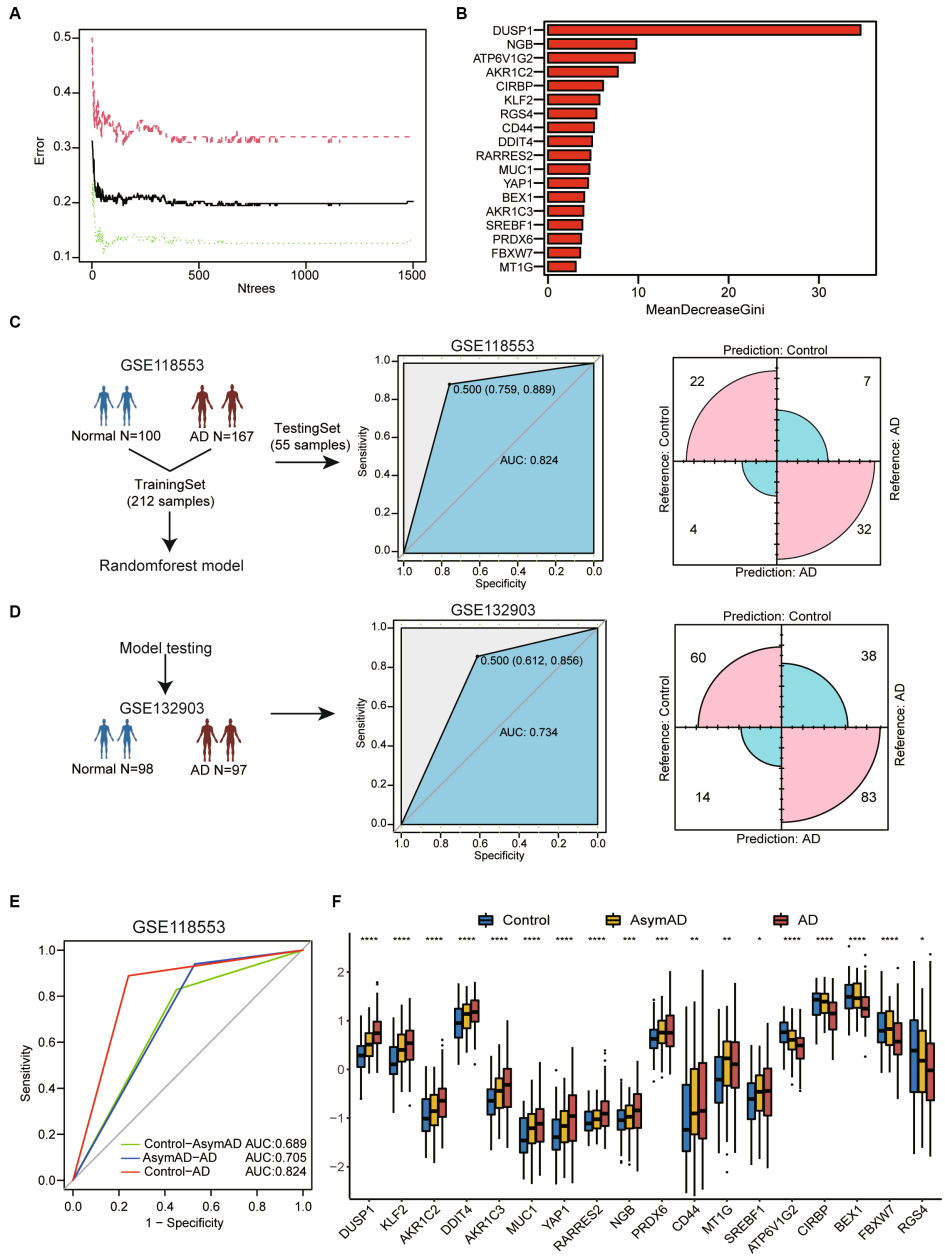
Machine-learning-based for constructing diagnostic model of AD. **(A)** The line chart shows the change in the number of decision trees and the error value of the model. When the number of decision trees is greater than 1,000, the error value tends to be stable. **(B)** The barplot shows importance of variables through MeanDecreaseGini coefficients. **(C)** The flow chart of random forest model construction through GSE118553. Receiver operating characteristic (ROC) curve for the testing model and confusion matrix of the hub genes combination in GSE118553. **(D)** The diagnostic model verification model in GSE132903. ROC curve for the testing model and confusion matrix in validation cohort. **(E)** ROC curve for the classification model between three cohorts. Calculation of AUC values in the cohorts by 5-fold cross-validation. **(F)** Boxplot shows 18 ferroptosis related genes expression in control, AsymAD and AD groups. *p < 0.05, **p < 0.01, ***p < 0.001, ****p < 0.0001.

To assess the potential of the above gene list for diagnosing AD, we constructed a RandomForest model. We performed model construction using 18 genes associated with ferroptosis and selected 1,000 as the number of decision trees in the Random Forest model (Figure 2A). The Gini coefficient indicated that these genes have high importance for predicting AD (Figure 2B). Firstly, we divided the 267 samples from GSE118553 into a training set and a testing set using a ratio of 4:1 (Figure 2C). The AUC value was 0.824 (95% confidence interval [CI] = 0.759–0.889) and corresponding matrix demonstrated that the training model could correctly classify AD and healthy samples with high accuracy. Then, we performed model testing using GSE132903 (Figure 2D). The AUC value and corresponding matrix demonstrated that the model achieved a high diagnostic accuracy in GSE132903 as well. Furthermore, we found that the model can also be used for the classification of asymptomatic AD (AsymAD). Compared to diagnosing healthy controls and AsymAD (AUC: 0.689), the model exhibited higher accuracy in diagnosing AsymAD and AD (AUC: 0.705) (Figure 2E).

We also discovered that these genes exhibit consistent expression trends in AsymAD and AD compare to healthy controls (Figure 2F). This may suggest that the hub genes related to ferroptosis may play an important role in the progression stages of the disease.

### Clinical and molecular characteristics of AsymAD and AD

**Figure 3:**
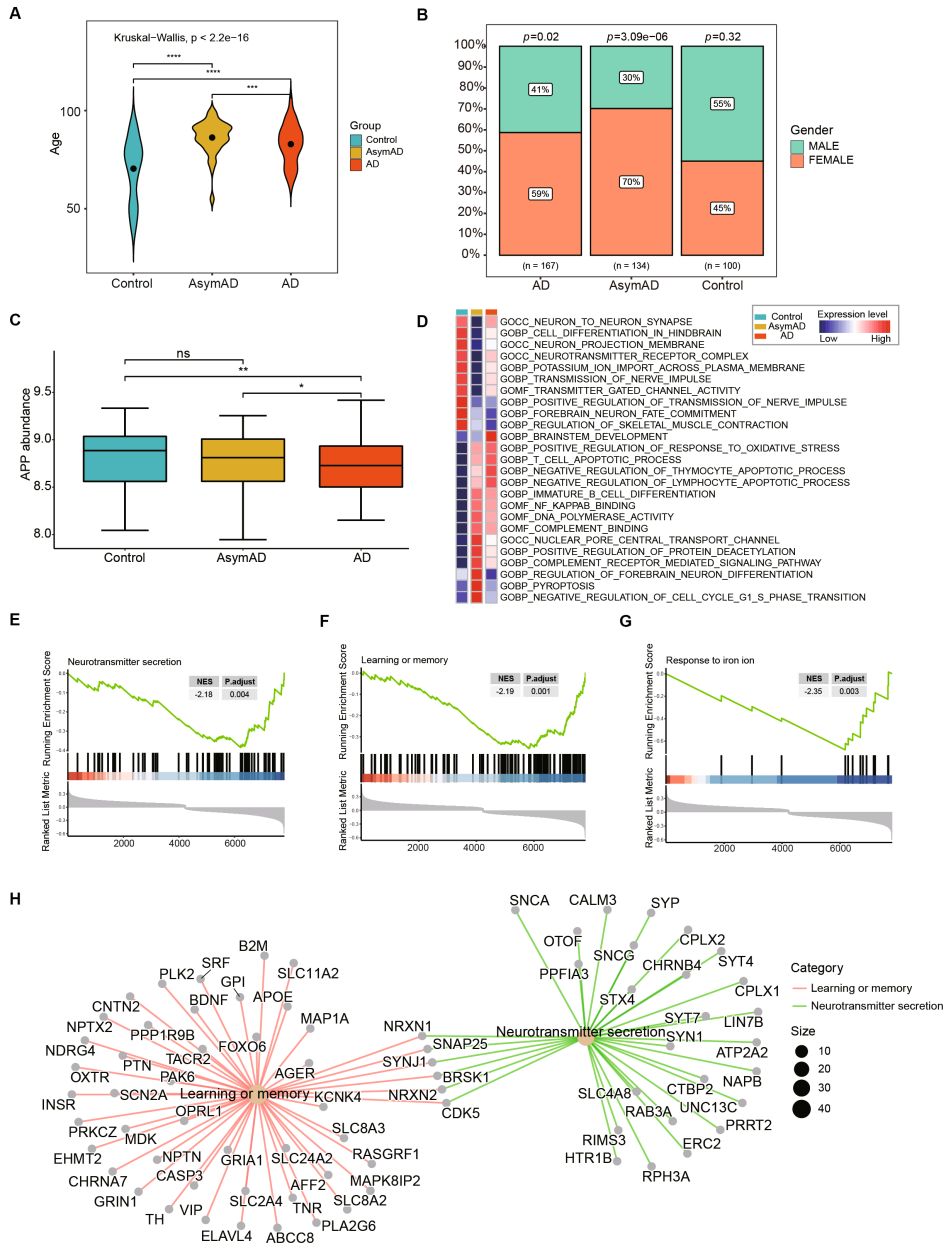
Clinical and molecular characteristics of AsymAD and AD. **(A)** The age difference between controls, AsymAD and AD patients. Data were analyzed by Kruskal−Wallis test. ***p < 0.001, ****p < 0.0001. **(B)** The difference of gender ratio in different groups. Data were analyzed by chi-squared test. **(C)** The boxplot shows APP expression in controls, AsymAD and AD groups. ns, not significant; *p < 0.05, **p < 0.01. **(D)** The heatmap plot shows pathway mean expression levels in different groups. The expression level of each pathway was determined by GSVA score. **(E–G)** The enrichscores and adjust pvalue of Neurotransmitter secretion, Learning or memory and Response to iron ion pathways. The results were calculated by GSEA analysis between AsymAD and AD groups. **(H)** The network plot shows the major down-regulated genes onvolved in pathway between AsymAD and AD groups.

We analyzed the demographic differences including age and gender between AsymAD and AD. The results indicated that there were significant differences in age among the control, AsymAD, and AD groups (Figure 3A). Studies have shown that there may be a gender difference in the prevalence and risk of AD (Masters et al., 2015). Women are more likely to develop AD than men and our results showed that there was a higher proportion of females than male (Figure 3B). We also observed that the expression level of APP AD was significantly lower than that of AsymAD, while there was no significant difference between AsymAD and control (Figure 3C). In contrast, in order to comprehend the potential biological functions difference between AsymAD and AD, enrichment analyses were conducted. The GSVA results revealed that the expression of oxidative stress response pathways was elevated in both AsymAD and AD (Supplementary Table 1), and the expression of Neuron-to-Neuron synapse and Nerve impulse pathways were decreased in AD compared to control and AsymAD, suggesting abnormalities in neurological related pathways in AD (Figure 3D). GSEA of the DEGs in AsymAD and AD has shown that they are associated with neurological related pathways include Neurotransmitter secretion and learning or memory (Figures 3E,F) and iron ion-related biological processes include Response to iron ion (Figures 3G,H). The results reveal significant demographic disparities, particularly in age and gender, between AsymAD and AD groups, and also underscore the complex interplay between demographic factors and molecular mechanisms in the progression of Alzheimer’s disease.

### Distinct brain regional molecular characteristics and gene expression patterns in AsymAD and AD

**Figure 4:**
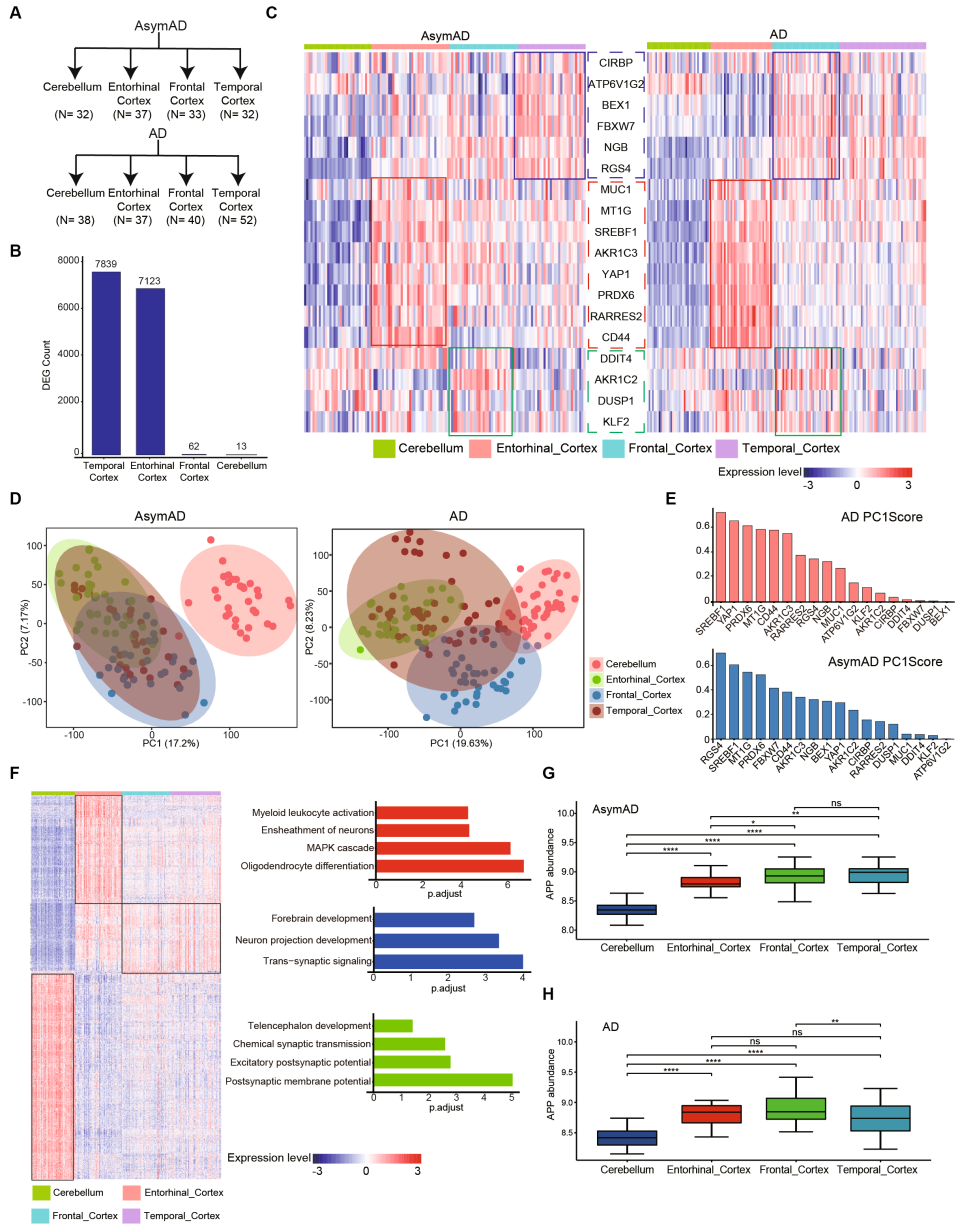
Molecular characteristics in different brain regions of AD and AsymAD. **(A)** The number of four brain regions (cerebellum, entorhinal, frontal and temporal cortex) in AsymAD and AD groups. **(B)** Barplot shows the contribution of DEGs (AsymAD and AD) from different brain regions. **(C)** Heatmap indicates ferroptosis-related genes specifically highly expressed in different brain regions. **(D)** Principal component analysis (PCA) showing two principal components of different regions in AsymAD and AD samples. **(E)** Barplot shows the score of genes with principal component 1 (PC1). Red bar represents AD, blue bar represents AsymAD. **(F)** Heatmap of specifically highly expressed genes in each brain region and the pathways in which they are involved. **(G,H)** The abundance of APP gene in four regions of AsymAD and AD. ns, not significant; *p < 0.05, **p < 0.01, ***p < 0.001, ****p < 0.0001.

To further investigate the connection between different brain regions and AD, we examined four regions in the brain, namely the Cerebellum, Entorhinal Cortex, Frontal Cortex, and Temporal Cortex, in both AD and AsymAD (Figure 4A). We observed that the Temporal Cortex and Entorhinal Cortex exhibited the most significant changes in both AD and AsymAD, suggesting that these two regions may play a crucial role in disease progression (Figure 4B). Further, we examined the expression of ferroptosis-related hub genes across different brain regions (Figure 4C). We found that MUC1, MT1G, SREBF1, AKR1C3, YAP1, PRDX6, RARRES2, and CD44 were specifically high-expressed in Entorhinal Cortex, and this trend was more pronounced in AD. Similarly, DDIT4, AKR1C2, DUSP1, and KLF2 were highly expressed in the Frontal Cortex of both AD and AsymAD. CIRBP, ATP6V1G2, BEX1, FBXW7, NGB, and RGS4 were highly expressed in the Temporal Cortex of AsymAD, but were highly expressed in the Frontal Cortex of AD. These findings suggest region-specific roles of these genes in the pathogenesis of AD and AsymAD.

The results of principal component analysis (PCA) revealed distinct differences between the Cerebellum and other regions in patients AsymAD (Figure 4D). On the other hand, the distribution of the Entorhinal Cortex, Frontal Cortex, and Temporal Cortex appeared to be more similar, indicating a similarity in molecular characteristics among these three regions in AsymAD. In contrast, the molecular characteristics of the four regions showed significant changes in AD. By observing the score of gene with principal component 1 (PC1), we found that genes highly expressed in entorhinal cortex (SREBF1, PRDX6, CD44, MTIG and AKR1C3) had high scores with PC1 in both AsymAD and AD (Figure 4E). Furthermore, we conducted further analysis on region-specific genes present in both AsymAD and AD (Figure 4E). The results demonstrated that both the Cerebellum and Entorhinal Cortex exhibited distinct molecular features compared to other regions (Figure 4F). In the Entorhinal Cortex, region-specific genes are primarily involved in Myeloid leukocyte activation, Ensheathment of neurons, MAPK cascade, and Oligodendrocyte differentiation. In the Cerebellum, region-specific genes are mainly associated with Telencephalon development, Chemical synaptic transmission, Excitatory postsynaptic potential, and Postsynaptic membrane potential. Additionally, we identified a group of genes that are highly expressed in regions other than the Cerebellum. These genes are primarily involved in Forebrain development, Neuron projection development, and Trans-synaptic signaling. Additionally, we observed consistent downregulation of APP in both AsymAD and AD cerebellum (Figures 4G,H). Our findings shed light on the regional heterogeneity and molecular signatures associated with AD and AsymAD, providing valuable insights for further research and understanding of the disease.

### Immune microenvironment alterations in AD

**Figure 5:**
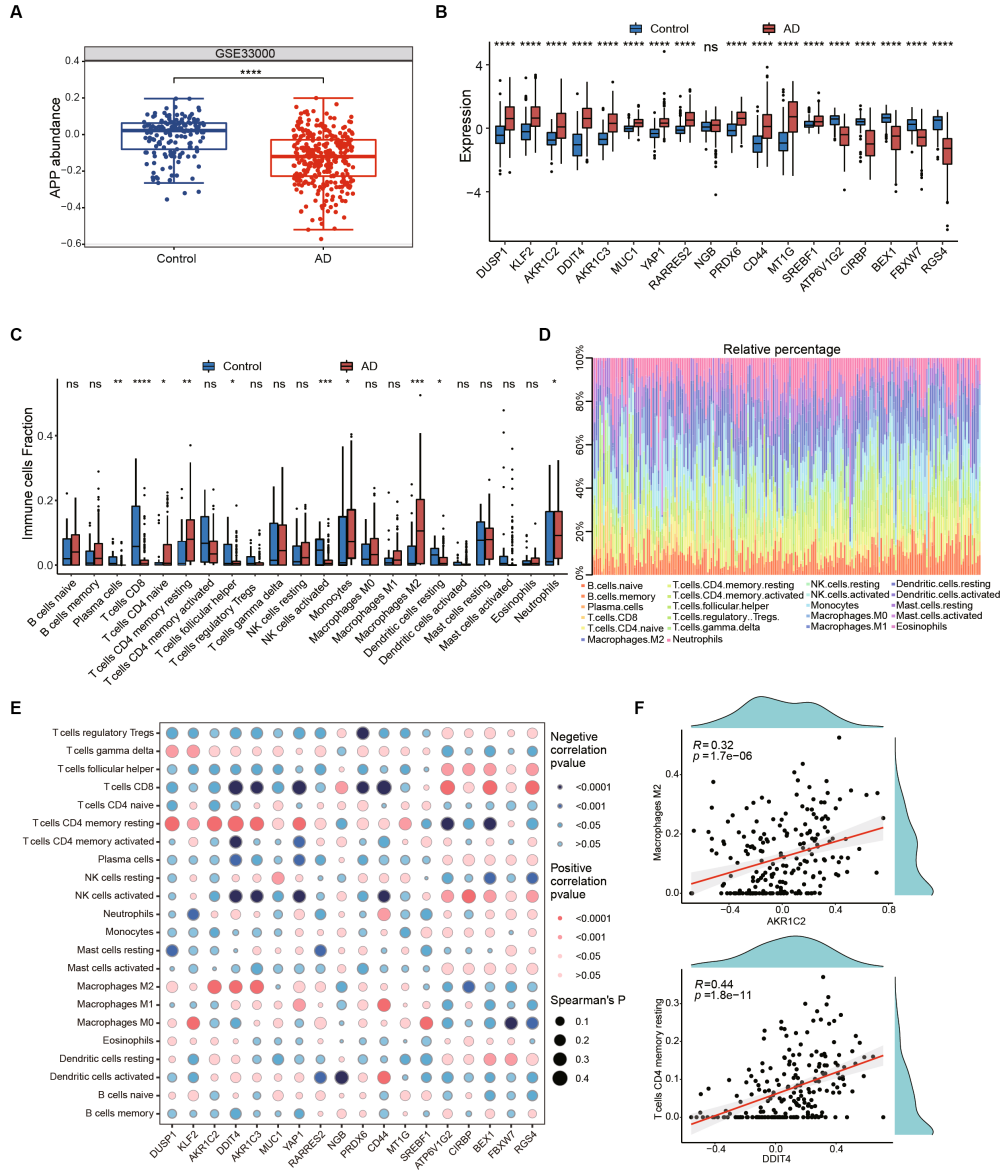
Immune infiltration and immune-related factors. **(A)** The boxplot shows the expression of APP in control and AD groups. ****p < 0.0001. **(B)** The boxplot shows expression of ferroptosis related genes in GSE33000 including healthy controls and AD groups. Ns, not significant; *p < 0.05, **p < 0.01, ***p < 0.001, ****p < 0.0001. **(C)** Boxplots showed the differences in immune infiltration between control, HD and AD. **(D)** The relative abundances of 22 infiltrated immune cells among control, HD and AD. **(E)** Correlation dotplot shows correlation between genes and immune cell infiltration. Red dot represents positive correlation while blue dot represents negative. The size of the points are determined by the value of Spearman’s coefficient. **(F)** Scatter plots showed the correlation between AKR1C2 and M2 macrophages, DDIT4 and T cells CD4 memory resting.

The clinical therapeutic sensitivity and disease diagnosis are significantly influenced by the microenvironment, which comprises immune cells, extracellular matrix, inflammatory factors, and a diverse range of growth factors. To investigate the alterations in the immune microenvironment of AD patients, we employed the CIBERSORT algorithm to assess the proportions of immune cells in AD and healthy control groups in GSE33000. Firstly, we compared the expression of APP and ferroptosis-related genes in the GSE33000 dataset between control and AD groups. We found that APP was significantly downregulated in AD group (Figure 5A). Additionally, the expression patterns of ferroptosis-related hub genes were consistent with our previous findings (Figure 5B). The CIBERSORT algorithm results showed that CD4+ T cells naive, CD4+ T cells resting memory, macrophages M2 and neutrophils were significantly higher in AD. In contrast, plasma cells, CD8+ T cells, T cells follicular helper and NK cells activated were significantly lower in AD (Figures 5C,D). We subsequently investigated the correlation between hub genes and immune infiltration (Figure 5E). The hub genes were significantly negatively associated with CD4+ T cells resting memory, CD8+ T cells, macrophages M2, NK cells activated and neutrophils, which suggests that hub genes have a significant impact on the immune microenvironment. DDIT4 showed strong positive correlation with CD4 T cells memory resting and AKR1C2 had positive correlation with Macrophages M2 (Figure 5F). These results suggest that ferroptosis-related hub genes may play an important role in the immune microenvironment of AD patients.

### Potential therapeutic drug search based on ferroptosis-related hub genes

**Figure 6:**
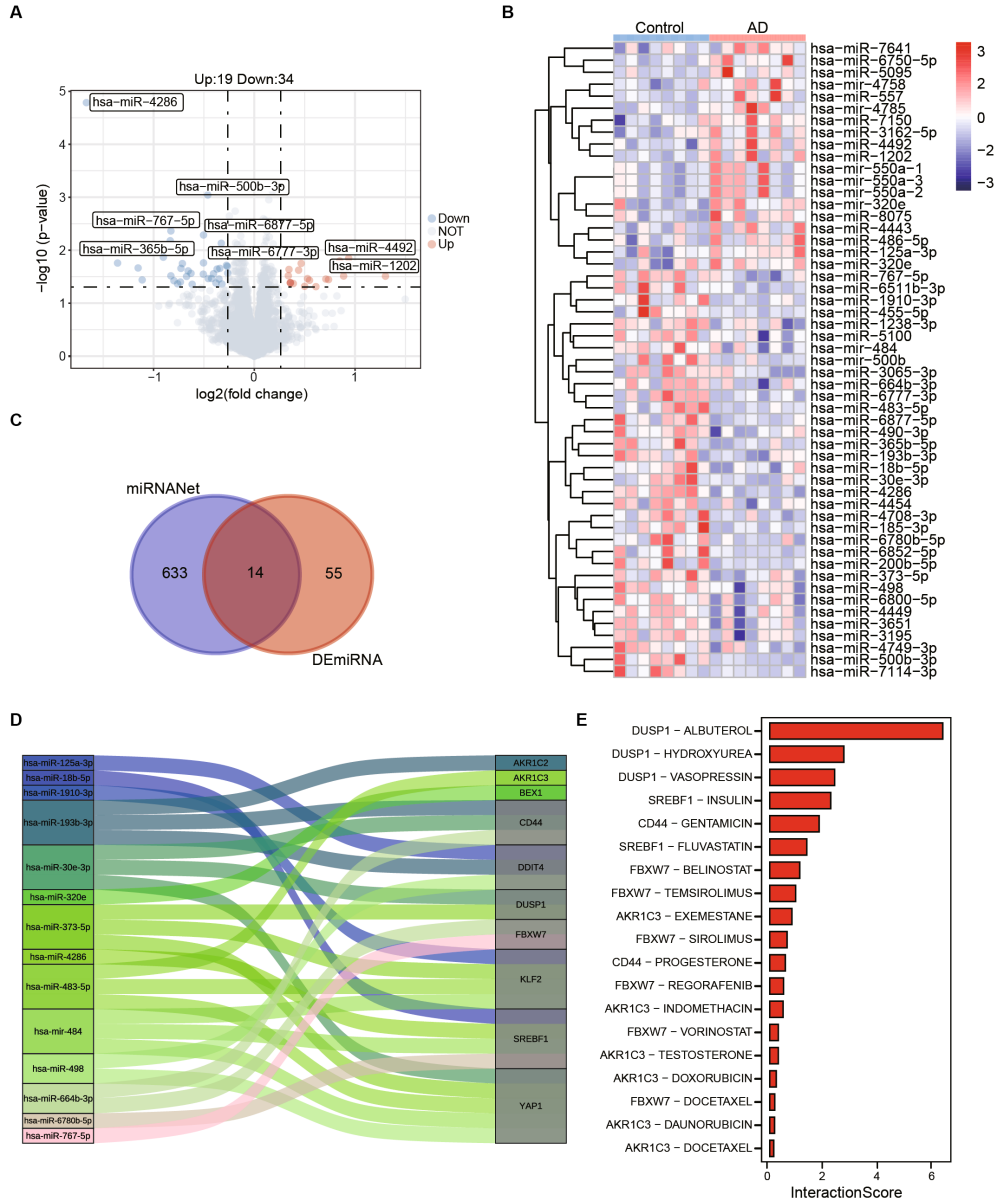
Potential therapeutic drug search based on ferroptosis-related hub genes. **(A)** The volcano map shows differentially expressed miRNAs between control and AD. **(B)** The abundances of 55 (Up:19, Down:34) differentially expressed miRNAs in control and AD patients. **(C)** A total of 633 miRNAs targeting ferroptosis related genes were obtained from miRNANnet. Fourteen kinds of miRNAs are obtained by intersected with differentially expressed miRNAs. **(D)** The Sankey plot shows 14 miRNAs and genes they target. **(E)** The interaction scores of genes and drugs were obtained by DGIdb.

In order to identify potential small molecule drugs for the treatment of AD, we analyzed 55 differentially expressed miRNAs (DEmiRNAs) including 19 up-regulated miRNAs and 34 up-regulated miRNAs between AD and healthy control groups using GSE157239 (Figures 6A,B). Subsequently, to further investigate whether these DEmiRNAs are associated with ferroptosis-related hub genes, we utilized the miRnet database and finally identified 14 miRNAs that target ferroptosis-related hub genes as potential therapeutic targets (Figures 6C,D; Supplementary Table 2). Furthermore, we further confirmed the close association between AD and 7 miRNAs (hsa-miR-125a, hsa-miR-18b-5p, hsa-miR-193b, hsa-miR-373, hsa-miR-4286, hsa-miR-483, and hsa-miR-664b-3p) through the Human microRNA Disease Database (HMDD) (Table 2).

**Table 2:** Associations between microRNAs and AD.

miRNA name	Evidence code	Disease name	PMID	Description	Causality
hsa-mir-125a	genetics_knock down_promote	Alzheimer disease	32626959	Rescue experiments were conducted by transfecting lnc-ANRIL knockdown plasmid and lnc-ANRIL knockdown plasmid and miR-125a inhibitor in the PC12 cellular AD model as the KD-ANRIL group or KD-ANRIL KD-miR-125a group, respectively. Lnc-ANRIL knockdown suppressed cell apoptosis and inflammation while promoting neurite outgrowth *via* binding of miR-125a in AD.	Unknown
hsa-mir-18b	other	Alzheimer disease	36723144	Taking miR-18b-5p and miR3p which are associated with Alzheimer’s disease as an example, a FRET sensing system was fabricated for the simultaneous analysis of two miRNAs within 1 h at picomolar concentration.	Unknown
hsa-mir-193b	circulation_biomarker_diagnosis_ns	Alzheimer disease	25119742	MicroRNA-193b is a regulator of amyloid precursor protein in the blood and cerebrospinal fluid derived exosomal microRNA-193b is a biomarker of Alzheimer’s disease.	NO
hsa-mir-373	circulation_biomarker_diagnosis_up	Alzheimer disease	36422510	Therefore, we suggest that miR-204 and miR-373 are potential biomarkers for AD.	Unknown
hsa-mir-4286	tissue_expression_down	Alzheimer disease	32920076	The analyses yielded 6 miRNAs differentially expressed	Unknown
hsa-mir-483	circulation_biomarker_diagnosis_ns	Alzheimer disease	24577456	Genome-wide serum microRNA expression profiling identifies serum biomarkers for Alzheimer’s disease.	NO
hsa-mir-664b	Other	Alzheimer disease	36153426	Moreover, two miRNAs (miR-3651, miR-664b-3p) showed significant differential expression in AD brains versus controls, in accordance with the change direction of lead exposure.	Unknown

To further refine our search, we employed the Drug-Gene Interaction Database (DGIdb) to identify compounds with high interaction scores with the hub genes (Figure 6E; Supplementary Table 3). These findings suggest that these miRNAs and drugs may hold promise as potential therapeutic agents for AD, and further investigation is warranted to determine their efficacy and safety.

## Discussion

Ferroptosis is a form of regulated cell death characterized by the accumulation of iron and lipid peroxidation, leading to cell membrane damage and ultimately cell death. In the context of AD, dysregulation of iron metabolism and increased oxidative stress contribute to the accumulation of toxic protein aggregates, such as amyloid-β plaques and tau tangles. This exacerbates neurodegeneration and cognitive decline in Alzheimer’s patients. In addition, a substantial proportion from 20 to 30%, of cognitively intact elderly individuals exhibit amyloid-β protein accumulation. Compared to those without amyloid-β, these individuals have a higher risk of progressing to AD and are commonly referred to as AsymAD individuals (Driscoll and Troncoso, 2011).

In this study, we identified differentially expressed genes associated with ferroptosis in AD patients using the GSE118553. We further validated the expression trends of these hub genes using the GSE132903, demonstrating consistency in their expression patterns. Among the ferroptosis-related hub genes we identified, four genes were involved in driving ferroptosis: KLF2, YAP1, CIRBP, and FBXW7. KLF2 has been found to have a negative regulatory relationship with APP and overexpression of KLF2 attenuated Aβ-induced oxidative stress (Wu et al., 2013; Fang et al., 2017). YAP1 is a transcriptional co-activator that has been linked to neurodegenerative diseases, including AD (Xu et al., 2021). CIRBP demonstrated neuroprotective effects against amyloid-induced neuronal toxicity through antioxidative and antiapoptotic pathways (Su et al., 2020). FBXW7 is a ubiquitin ligase that plays a role in protein degradation and has been reported that plays a crucial role in neurological functions, particularly in neurodevelopment and the pathogenesis of neurodegeneration (Yang et al., 2021). Furthermore, we identified that 9 DEGs are involved in suppressing ferroptosis process. AKR1C2 and AKR1C3 belong to the aldo-keto reductase family, which are involved in the metabolism of steroid hormones and other endogenous compounds. Dysregulation of AKR1C2 and AKR1C3 has been observed in Alzheimer’s disease (Luchetti et al., 2011; Chik et al., 2022), suggesting their potential role in disease progression. PRDX6 has already been reported to be involved in the oxidative stress and antioxidant defense process in AD (Viejo et al., 2022). CD44 is a cell surface glycoprotein and involve in the neuroinflammatory processes mediated by microglial cells (Rangaraju et al., 2018; Smajić et al., 2022). SREBF1 has also been confirmed as a disease-associated transcription factor (Morabito et al., 2021). BEX1 has been reported to potentially be associated with the gender disparity in AD (Garcia et al., 2023). However, the relationship between MUC1, RARRES2 and MT1G with the disease is not extensively studied, and the specific role of them in AD pathogenesis is still being elucidated. In addition, we have also identified 5 unclassified ferroptosis-related genes that are associated with AD: DUSP1, DDIT4, NGB, ATP6V1G2, RGS4. There have been reports indicating a significant negative correlation between DUSP1 and cognitive abilities in AD patients (Qi et al., 2022). In the context of AD, DDIT4 responds to extracellular amyloid-β and regulates the cytotoxic effects of amyloid-β *in vitro* (Morel et al., 2009). NGB is a neuroglobin protein, belonging to the globin family. The overexpression of NGB can protect neurons from mitochondrial dysfunction and neurodegenerative diseases such as AD (Ascenzi et al., 2016). ATP6V1G2, a subunit of the vacuolar ATPase (V-ATPase) complex. Studies have shown that ATP6V1G2 as significantly regulated by DNA methylation at hub CpGs in AD (Kim et al., 2023). RGS4, a regulator of G-protein signaling, has been identified as a potential biomarker for AD in multiple studies (Zou et al., 2019; Chen et al., 2022), and this finding has been confirmed in our result as well. Although these genes exhibit consistent strong correlations in both datasets, there are some genes whose correlations show completely opposite patterns between the two datasets. Therefore, further investigation into the stability of interactions between ferroptosis-related hub genes may be necessary to provide additional evidence. This could be an important aspect to consider in future research.

To investigate the potential of these genes as biomarkers for AD, we constructed a random forest classifier model. Our results showed that this model was able to accurately distinguish between healthy controls and AD patients in both the training sets (GSE118553, AUC = 0.824) and validation sets (GSE132903, AUC = 0.734). Furthermore, we found that the model was also able to differentiate between AsymAD and control groups (AUC = 0.689), and also can distinguish between AsymAD and AD (AUC = 0.705), indicating that these genes may play an important role in the progression of AD. Demographic characteristics also indicated that both AsymAD and AD patients had significantly higher age compared to the healthy control group. Additionally, a higher proportion of females was observed in both AsymAD and AD patients, which is consistent with previous research findings. Furthermore, we discovered significant downregulation of pathways related to learning or memory, neurotransmitter secretion, and response to iron ion in AD patients compared to AsymAD patients. Besides, in our analysis of genes involved in the learning or memory pathways, we discovered that APOE and AGER exhibit dysregulated expression in AD. APOE is involved in lipid metabolism and is a major genetic risk factor for late-onset Alzheimer’s disease, with the APOE ε4 allele being particularly associated with an increased risk (Serrano-Pozo et al., 2021; Martens et al., 2022). AGER is involved in the regulation of inflammation and oxidative stress. In Alzheimer’s disease, AGER contributes to the accumulation of amyloid-beta plaques and the activation of microglia, which can exacerbate neuroinflammation and neuronal damage (Ding et al., 2020).

Further, we observed the transcriptional expression profiles of different brain regions in AsymAD and AD, respectively. By assessing the contribution of different brain regions in DEGs of AsymAD and AD, we found that the DEGs of AsymAD and AD were mostly from temporal cortex and frontal cortex, which was consistent with previous studies (Patel et al., 2019). In different regions of AsymAD and AD, we found MUC1, MT1G, SREBF1, AKR1C3, YAP1, PRDX6, RARRES2 and CD44 were upregulated in both AsymAD and AD entorhinal cortex region. DDIT4, AKR1C2, DUSP1 and KLF2 were all upregulated in frontal cortex region. Notably, CIRBP, ATP6V1G2, BEX1, FBXW7, NGB, and RGS4 were upregulated in AsymAD temporal cortex region but upregulated in AD frontal cortex region. These results indicate that ferroptosis-related hub genes can reflect the transcriptional changes of different brain regions in pathological states. It is worth noting that the changes of ferroptosis-related hub genes in other brain regions, such as the hippocampus, which have a significant impact on AsymAD or AD, have not been elucidated in our study. This is a crucial aspect that requires further research and validation in the future. We also identified specific high-expression genes in different brain regions. Entorhinal cortex specific high expression genes are mainly related to myeloid leukocyte activation, ensheathment of neurons, MAPK cascade and oligodendrocyte differentiation. The highly expressed genes of cerebellum are mainly involved in telencephalon development, chemical synaptic transmission, excitatory postsynaptic potential and postsynaptic membrane potential. We also identified a group of genes that were highly expressed in regions other than the cerebellum, which primarily associated with forebrain development, neuron projection development, and trans−synaptic signaling.

Studies have shown that both microglia and astrocytes, as well as peripheral immune cells, are involved in neuroinflammation associated with AD (Leng and Edison, 2021). Therefore, we investigated immune infiltration in AD, we found that M2 macrophages’ infiltration was significantly increased in AD, which is consistent with previous study (Lin et al., 2022). In addition, neutrophils and monocytes were also upregulated in AD. In contrast, CD4 T cells memory resting was downregulated. Further, we found ferroptosis-related hub genes was highly correlated with immune cell infiltrations. DDIT4 showed strong positive correlation with CD4 T cells memory resting and AKR1C2 had positive correlation with Macrophages M2. It has been shown that downregulation of neuronal DDIT4 can restore the proliferative characteristics of glial cells and the abnormal expression of key proteins of inflammasome (Pérez-Sisqués et al., 2021). Therefore, studying the role of immune and inflammatory cells in AD may provide anti-inflammatory and immunomodulatory targets for AD treatment.

Here, miRNAs targeting ferroptosis related hub genes were identified by miRNAnet. We identified 14 miRNAs which targeting 10 of ferroptosis related genes. miR-125a showed promise in regulating cell functions and inflammation in diseases associated with neuronal dysfunction (Potenza and Russo, 2013). In addition, study also reported that in a rat model of ethanol-induced neurotoxicity, the SVCT2 mitigated oxidative injury by modulating the JNK/p38 MAPK, NF-κB, and miR-125a-5p pathways (Tian et al., 2016). Moreover, it has been observed that miR-483-5p plays a role in repressing the activity of ERK1/2, leading to a decrease in the phosphorylation of TAU protein at epitopes associated with TAU neurofibrillary pathology in AD (Nagaraj et al., 2021). Then, 19 drugs targeting the above genes were retrieved from the DGIdb database. Albuterol also known as Salbutamol, is a short-acting β 2-adrenoceptor agonist. Increased amyloid β production after β 2-adrenoreceptor activation was reported by Ni et al. (2006), animal models of memory disruption have shown improved performance after Albuterol treatment (Ciprés-Flores et al., 2019). In our results, Albuterol showed the highest interaction score with DUSP1, indicating that DUSP1 may be a promising drug target. Insulin plays an important role in the regulation of glucose metabolism and can influences cerebral bioenergetics, turnover of neurotransmitters in AD (Arnold et al., 2018; Kellar and Craft, 2020). A study has reported Hydroxyurea provides neuroprotection *in vitro* against neurotoxins, which increase oxidative stress and excitotoxicity and reduce mitochondrial function (Brose et al., 2018).

## Conclusion

In conclusion, we acquired 18 ferroptosis-related hub genes in AD. We explored the potential of these genes as diagnostic markers and their role in the disease process which will help in understanding the development of AD. However, further experimental validation is needed to verify their functions. In addition, these hub genes have high correlation with infiltration of immune cells. Currently, a few drugs targeting these hub genes are predicted to relieve AD, provides new research clues for preventing the development of AD. Our study also has limitations, we used data from public databases for our analysis, which were from different platforms. Due to different sequencing technologies and platforms, the inclusion criteria of patients are different. Furthermore, our study is limited to the transcriptome level, the significance of these findings requires further validation through prospective clinical and basic experiments.

## Data availability statement

The original contributions presented in the study are included in the article/Supplementary material, further inquiries can be directed to the corresponding authors.

## Author contributions

HZ: Writing – original draft. JW: Writing – original draft. ZL: Formal analysis, Writing – review & editing. SW: Visualization, Writing – review & editing. GY: Methodology, Writing – original draft, Writing – review & editing. LW: Writing – original draft.

## Footnotes

^1^ https://www.ncbi.nlm.nih.gov/geo/

## Supplementary material

SUPPLEMENTARY FIGURE 1 **(A)** Volcano plot of differentially expressed genes differing significantly between healthy and AD samples in GSE118553. **(B)** Volcano plot of differentially expressed genes differing significantly between healthy and AD samples in GSE132903. **(C)** The boxplot shows expression of ferroptosis related genes in GSE132903 including healthy controls and AD groups. **p* < 0.05, ***p* < 0.01, ****p* < 0.001, *****p* < 0.0001.
