# Supplementary figures and images for: Identification ferroptosis-related hub genes and diagnostic model in Alzheimer’s disease
Source: Front Mol Neurosci. 2023 Oct 30;16:1280639. doi: 10.3389/fnmol.2023.1280639 (PMC10642492; doi:10.3389/fnmol.2023.1280639)

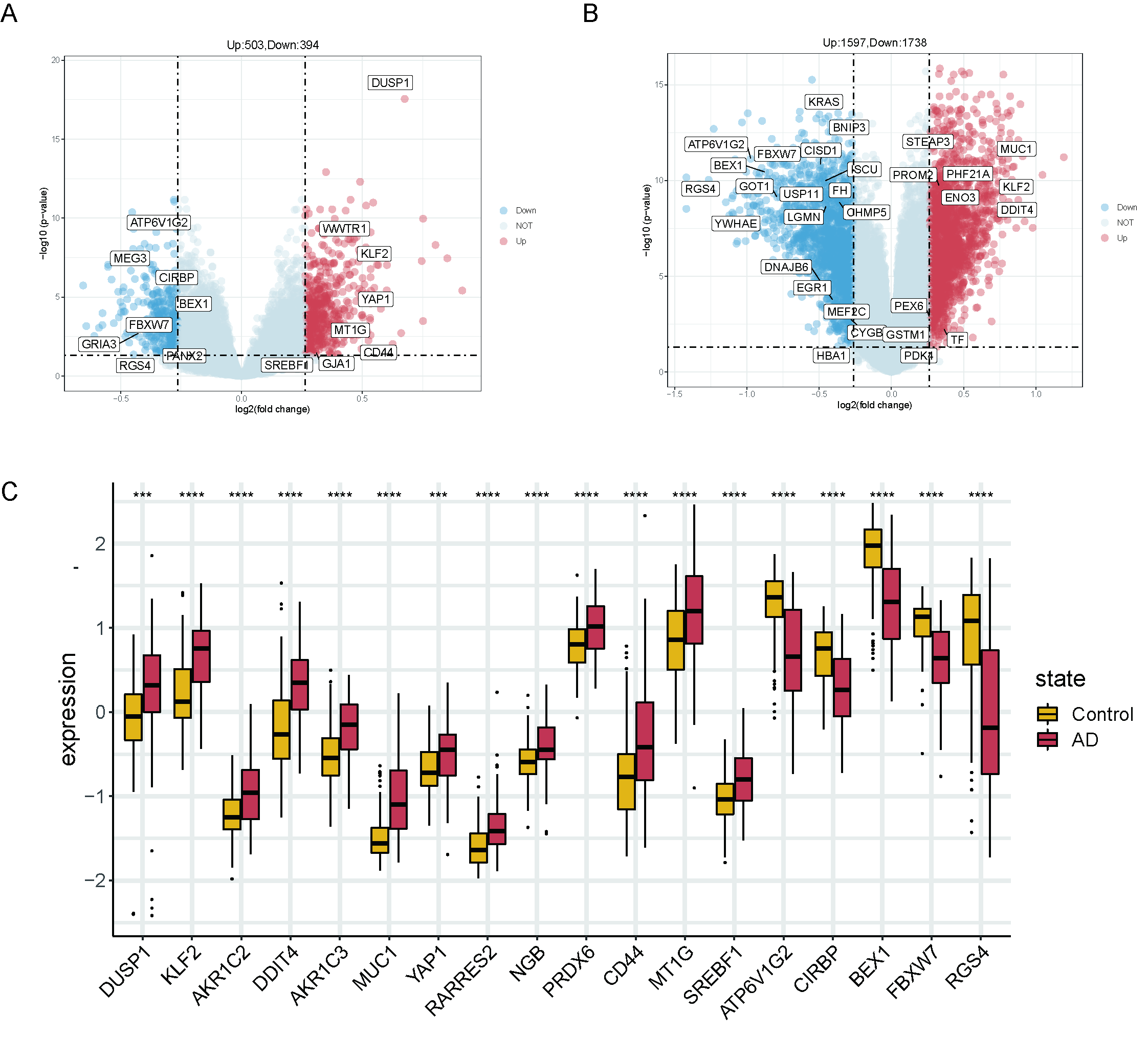

Supplement: SUPPLEMENTARY FIGURE 1 — (A) Volcano plot of differentially expressed genes differing significantly between healthy and AD samples in GSE118553. (B) Volcano plot of differentially expressed genes differing significantly between healthy and AD samples in GSE132903. (C) The boxplot shows expression of ferroptosis related genes in GSE132903 including healthy controls and AD groups. *p < 0.05, **p < 0.01, ***p < 0.001, ****p < 0.0001. [file Image_1.tif]
